# Relationship between TyG index and the degree of coronary artery lesions in patients with H-type hypertension

**DOI:** 10.1186/s12933-023-02013-0

**Published:** 2024-01-12

**Authors:** Zhengwen Xu, Peixian Chen, Lian Wang, Jie Yan, Xisheng Yan, Dongsheng Li

**Affiliations:** 1 Department of Cardiology, Wuhan Third Hospital & Tongren Hospital of Wuhan University, Wuhan, 430074 Hubei China; 2 Department of Forensic Science, School of Basic Medical Science, Central South University, Changsha, 410013 Hunan China

**Keywords:** Coronary artery disease, Degree of coronary stenosis, Triglyceride glucose index, H-type hypertension

## Abstract

**Background:**

The TyG index, a prominent metric for assessing insulin resistance, has gained traction as a prognostic tool for cardiovascular disease. Nevertheless, the understanding of the prognostic significance of the extent of coronary artery stenosis in individuals afflicted with H-type hypertension remains limited.

**Methods:**

A retrospective study was conducted at Wuhan Third Hospital, including a cohort of 320 inpatients who were diagnosed with hypertension in combination with coronary artery disease. The study period spanned from January 1, 2021, to February 1, 2023. The study cohort was stratified based on the severity of stenosis into three distinct groups: low stenosis, medium stenosis, and high stenosis, as determined by the Gensini score derived from coronary angiography findings. The present study aimed to investigate the association between the severity of coronary stenosis and the number of lesion branches, utilizing the TyG index as a testing indicator. The predictive ability of TyG for coronary lesion severity was assessed using logistic regression analysis.

**Results:**

The results of our study indicate a positive correlation between elevated levels of TyG and an increased susceptibility to severe stenosis in individuals diagnosed with H-type hypertension. Upon careful consideration of potential confounding variables, it has been observed that the TyG index exhibits a robust association with the likelihood of severe stenosis in individuals with H-type hypertension (odds ratio [OR] = 4000, 95% confidence interval CI 2.411–6.635, p = 0.0001), as well as the prevalence of multivessel disease (OR = 1.862, 95% CI 1.036–3.348, p < 0.0001). The TyG index demonstrated superior predictive ability for severe coronary stenosis in patients with H-type hypertension compared to those without H-type hypertension (area under the curve [AUC] = 0.888, 95% confidence interval CI 0.838–0.939, p < 0.0001, versus AUC = 0.615, 95% CI 0.494–0.737, p < 0.05).

**Conclusion:**

The TyG index is an independent risk factor for the degree of coronary stenosis and a better predictor in patients with H-type hypertension combined with coronary artery disease.

## Background

Cardiovascular disease (CVD) is the leading cause of mortality worldwide, with China experiencing its devastating effects as the primary cause of death and premature mortality [1]. The country is currently facing an urgent need to effectively address the increasing prevalence of CVD and the concurrent rise in patients suffering from coronary artery disease (CAD) [2].

China, a nation with a substantial population and a high prevalence of hypertension, has an estimated hypertensive population of no less than 200 million, based on comprehensive survey findings [3, 4]. Among this cohort, a substantial proportion, ranging from 68.3% to 80%, is diagnosed with H-type hypertension [5, 6]. H-type hypertension, a disorder where essential hypertension coexists with hyperhomocysteinaemia, has been identified [7]. The prevalence of cardiovascular events in patients with H-type hypertension is approximately five times greater compared to patients with hypertension alone and about 25–30 times greater compared to the general population [8]. The incidence of acute myocardial infarction is significantly higher in individuals diagnosed with H-type hypertension compared to those with uncomplicated hypertension. Moreover, concentrations of Hcy are significantly elevated in patients experiencing acute myocardial infarction as opposed to those who do not exhibit this condition [9], Moreover, It is worth noting that heightened Hcy levels are frequently linked to the presence of multiple vasculopathies [10]. Individuals diagnosed with H-type hypertension are subject to a considerably elevated likelihood of experiencing an unfavorable prognosis, primarily due to the diffuse and unstable characteristics exhibited by atherosclerotic plaques [11, 12]. There is a notable and statistically significant correlation between hypertension and insulin resistance [13], especially among patients with H-type hypertension [14]. The hyperglycemia and dyslipidemia resulting from IR act synergistically with elevated blood pressure, leading to the onset and progression of cardiovascular disease [15].

Insulin resistance (IR), denoting diminished sensitivity and responsiveness to the physiological actions of insulin, has been duly acknowledged as a defining feature of type 2 diabetes [16]. In typical circumstances, the presence of insulin at physiological levels induces vasodilation and enhances vascular recovery through the augmentation of nitric oxide (NO) synthesis by endothelial cells (ECs) [17, 18], However, in the context of insulin resistance (IR), these beneficial effects of insulin may be diminished, and in fact, insulin may elicit vasoconstriction by promoting the production of vasoconstrictive agents such as endothelin and/or contributing to the development of pathological atherosclerosis. Metabolic syndrome, otherwise delineated as an agglomeration of metabolic aberrations, including dysglycemia, dyslipidemia, and hypertension [19]. These aberrations have been firmly linked to an unfavorable prognosis for cardiovascular disease (CVD) [17]. Such association between metabolic syndrome and cardiovascular disease has become not only conspicuous but also vigorous [20]. the previous study unveil a robust, statistically significant correlation between insulin resistance (IR) and the risk of cardiovascular disease within a distinct group of individuals, specifically, those diagnosed with type 2 diabetes and suffering from insulin-resistant hypertension [21]. A more effectual and uncomplicated index to measure insulin resistance and thus gauge cardiometabolic risk is METS-IR. It outstrips traditional obesity indices in prognosticating hypertension and MetS [22]. The intricate nexus between the onset and progression of cardiovascular disease and METS-IR, an evaluative biological index for insulin resistance, has been recently substantiated [23, 24]. Moreover, the triglyceride-glucose index, often referred to as the TyG index, is gaining recognition as a reliable alternative biomarker for insulin resistance (IR). A substantial body of research has furnished persuasive empirical data fortifying the relationship between the TyG index and the genesis and prognosis of cardiovascular disease [25, 26].

Coronary artery angiography, known as the definitive diagnostic modality for coronary heart disease, exhibits a relatively low prevalence rate within primary healthcare facilities in China. This discrepancy primarily stems from the specialized knowledge and intricate procedural techniques required, as well as the invasive nature of the test. Currently, a notable absence exists in the realm of medical research regarding the availability of a biologically sound index that possesses a commendable level of specificity for evaluating the potential risks faced by patients afflicted with H-type hypertension in conjunction with coronary artery disease. Hence, it would be deemed a pioneering concept for the vast majority of primary care physicians and clinicians, who lack specialized expertise in cardiology, to effectively discern patients afflicted with coronary artery disease, particularly those presenting with more advanced stages of the condition, through a streamlined and easily accessible approach.

## Methods

### Study population and design

The present investigation was carried out as a cross-sectional observational study, adhering to the principles outlined in the Declaration of Helsinki. It is important to note that no data pertaining to patient privacy or identifiable attributes were collected for the purposes of this study. Furthermore, it is worth mentioning that the study protocol received approval from the Ethical Review Committee of Wuhan Third Hospital, located in the People's Republic of China. In accordance with ethical guidelines, written consent was duly obtained from all patients, ensuring that they were fully informed about the nature and purpose of the study, as well as the potential risks and benefits associated with their participation.

The present study recruited individuals who were admitted to the Department of Cardiology at the Third Hospital of Wuhan City and underwent coronary angiography between the period of January 2021 and January 2023. The inclusion criteria for this study encompassed the following parameters: (1) Participants were required to be adults aged 18 years or older; (2) The diagnostic criteria for hypertension included a documented history of hypertension, current use of antihypertensive medication, or systolic blood pressure (SBP) ≥ 140 mmHg or diastolic blood pressure (DBP) ≥ 90 mmHg; (3) The diagnostic criteria used to identify metabolic syndrome were based on the NCEP-ATP III criteria:fasting blood glucose ≥ 100 mg/dl, SBP or DBP ≥ 130 or ≥ 85 mmHg; HDL-C < 50 mg/dl for women or < 40 mg/dl for men; triglyceride ≥ 150 mg/dl; and waist circumference ≥ 80 cm for women or ≥ 94 cm for men) [27]; (4) The subjects with ≥ 50% stenosis in at least one main stem lumen were diagnosed with CAD [28]; (5) All participants underwent coronary angiography as part of the study protocol. The exclusion criteria encompassed the following: (1) Patients presenting contraindications to coronary angiography or an inability to cooperate with vascular function tests; (2) Patients with concurrent acute infection, severe arrhythmia, pregnancy or lactation, as well as severe hematological and endocrine system diseases; (3) Patients with incomplete clinical data and coronary angiography. In recent times, there has been a notable utilization of pharmaceutical interventions centered around folic acid.

### Data collection and grouping

The fundamental clinical data for each patient, encompassing sex, age, height, weight, heart rate, systolic blood pressure, diastolic blood pressure, as well as pertinent medical history such as hypertension, diabetes mellitus, atrial fibrillation, smoking history, and drug use history, was meticulously extracted from the patient's medical record by a proficient clinician with specialized training. The blood pressure was determined as the mean value of three consecutive measurements obtained from the patient's arm, utilizing an electronic sphygmomanometer, under the supervision of a skilled clinician. Prior to the assessment, the patient was instructed to observe a period of tranquil rest lasting no less than 10 min. The calculation of the body mass index (BMI) involves dividing an individual's weight by the square of their height.

Blood samples were collected from the antecubital vein of fasting patients in the early morning for the purpose of laboratory test indications. The concentration of fasting plasma glucose (FPG) was determined using the hexokinase method. The levels of TGs, TC, HDL-C, and LDL-C, as well as the counts of leukocytes, neutrophils, and platelets, were quantified using the enzymatic method. Additionally, the concentrations of albumin, uric acid, and creatinine were determined using the same enzymatic approach. Furthermore, coronary angiography data were obtained and analyzed. The diagnostic criteria for H-type hypertension entail the inclusion of hypertensive patients who exhibit elevated plasma homocysteine concentrations of 15 mol/L [29] the TyG index was calculated as = Ln[TG(mg/dL) × FBG (mg/dL)/2] [30]. METS-IR is calculated as = (Ln [(2 × FPG) + TG] × BMI)/(Ln[HDL-C]) [23]. The Gensini score, on the other hand, was determined by a skilled cardiovascular physician who evaluated the patient's coronary angiographic results.

Based on the TyG index level, the study participants were categorized into four quartiles: Q1 (n = 80, TyG index ≤ 8.38), Q2 (n = 81, 8.38 < TyG index ≤ 8.88), Q3 (n = 79, 8.88 < TyG index ≤ 9.42), and Q4 (n = 80, TyG index ≥ 9.42). The study cohort was stratified into three distinct categories of stenosis severity, namely mild stenosis, moderate stenosis, and severe stenosis, employing a ternary approach based on the Gensini score. Specifically, individuals with a Gensini score of ≤ 32 were classified as belonging to the mild stenosis group (n = 114), those with a score ranging from 32 to 53 points were categorized as having moderate stenosis (n = 100), and participants with a score of ≥ 53 were assigned to the severe stenosis group (n = 106). Subsequently, the study cohort was stratified based on the Gensini score of the individuals. Those with a Gensini score exceeding 53 were categorized as having severe stenosis, comprising a total of 106 subjects. Conversely, individuals with a Gensini score equal to or below 53 were classified as not having severe stenosis, amounting to a population of 214 individuals.

### Statistical analysis

The determination of the sample size (n) was conducted utilizing the established formula: n = z^2^ p × (1−p)/e^2^. This calculation yielded a minimum sample size requirement of 317 patients, considering the estimated proportion of patients with severe coronary stenosis at 29% and a confidence level of 95%. The categorical variables within the baseline data of the study subjects were quantified in terms of numerical values and percentages. To evaluate the normality of the data, the Kolmogorov–Smirnov (K-S) test was employed. The means and standard deviations (SD) were calculated for continuous variables that followed a normal distribution, while medians (interquartile range) were computed for variables that exhibited skewness. A unidirectional analysis of variance (ANOVA) or Kruskal–Wallis test was employed to assess the differences among groups with respect to quantitative variables. The chi-squared test was employed to conduct a comparative analysis of categorical variables across different groups. The statistical analysis employed to examine the associations between quantitative parameters involved the utilization of Pearson's correlation test. The identification of risk factors was accomplished through the utilization of multi-way logistic regression analysis. Furthermore, the accuracy of the TyG indices in detecting both metabolic syndrome and coronary stenosis was evaluated by means of ROC curve analysis. The area under the curve (AUCs) was employed as a metric to determine the predictive value of the TyG indices for both metabolic syndrome and coronary stenosis. All statistical tests conducted in this study were two-tailed and analyzed using the SPSS software version 25.0 (SPSS, Inc., Chicago, IL, USA). A p-value of 0.05 was deemed to possess statistical significance.

## Result

### Main clinical characteristics of the study population

A total of 320 patients were included in this study, comprising of individuals with H-type hypertension and coronary artery disease (CAD) (n = 156), as well as non-H-type hypertensive patients with CAD (n = 164). The average age of the participants was 66.8 ± 10.4 years. Among them, 162 (50.1%) were male, with an average body mass index (BMI) of 23.9 ± 3.2 kg/m^2^. The average pulse rate was 76.6 ± 14.0 beats per minute, while the average homocysteine level was 17.5 ± 7.6 units. Additionally, the average TyG index was found to be 8.95 ± 0.81.

### Clinical and biochemical data characteristics according to TyG index quartile grouping

The fundamental characteristics of the four groups are presented in Table 1. Significant differences were observed in various parameters including age, body mass index (BMI), diabetes mellitus (DM), white blood cell count (WBC), platelet count, albumin levels, glucose levels, triglyceride levels, high-density lipoprotein cholesterol (HDL-C) levels, low-density lipoprotein cholesterol (LDL-C) levels, urate levels, METS-IR index, Gensini score, number of vasculopathies, and the presence or absence of triple-vessel disease were higher in the group with a higher TyG (all P < 0.05). The TyG index exhibited a significant positive correlation with several physiological parameters including body mass index (BMI), systolic blood pressure (SBP), heart rate, platelet count, albumin levels, triglycerides (TG), total cholesterol (TC), low-density lipoprotein cholesterol (LDL-C), and uric acid (P < 0.05). The observed relationship exhibited a significant negative correlation with age (P < 0.05) and high-density lipoprotein (HDL) levels (P < 0.001). The prevalence of diabetes mellitus (DM) (P < 0.001), H-type hypertension (P < 0.05), triple vascular disease (P < 0.001), and the metabolic syndrome (P < 0.001) exhibited a greater occurrence among patients with an elevated TyG index.

**Table 1:** Baseline characteristics of 4 groups

Variable	Q1 (n = 81)	Q2 (n = 80)	Q3 (n = 79)	Q4 (n = 80)	P value
TyG index	8.00 ± 0.27	8.63 ± 0.14	9.14 ± 0.16	10.00 ± 0.55	< 0.001
Age, years	69.3 ± 8.9	65.3 ± 10.9	67.3 ± 9.5	65.15 ± 11.7	0.036
Male	33 (40.7)	44 (55.0)	40 (50.6)	45 (56.3)	0.188
BMI, kg/m2	22.7 ± 3.2	23.3 ± 2.9	24.6 ± 3.3	25.0 ± 2.7	< 0.001
SBP, mmHg	136.8 ± 25.5	140.9 ± 22.4	140.4 ± 21.8	141.2 ± 23.4	0.612
DBP, mmHg	80.5 ± 14.9	83.2 ± 14.6	82.0 ± 10.6	81.6 ± 11.6	0.613
Heart rate, bpm	73.3 ± 14.5	76.1 ± 14.3	78.1 ± 13.2	78.8 ± 13.8	0.064
Smoker	2 (2.5)	9 (11.3)	24 (30.4)	53 (66.3)	< 0.001
Drinker	0 (0)	2 (2.5)	3 (3.8)	23 (28.7)	< 0.001
H-type hypertension	40 (49.4)	28 (35.0)	40(50.6)	46 (57.5)	0.035
METS-IR index	32.0 ± 5.7	35.2 ± 5.3	39.2 ± 6.2	45.3 ± 7.3	< 0.001
Diabetes mellitus	13 (16.0)	21 (26.3)	25 (31.6)	36 (45.0)	0.001
Metabolic syndrome	0(0)	0(0)	3(3.8)	43 (53.8)	< 0.001
Biochemical indicators					
WBC, 10^9^/L	5.7 ± 1.9	6.3 ± 2.1	6.4 ± 2.1	7.1 ± 1.9	0.001
Neutrophil ratio	65.9 ± 9.8	65.9 ± 10.6	65.6 ± 11.0	67.7 ± 9.2	0.533
Platelet, 10^9^/L	199.5 ± 50.7	211.1 ± 61.1	227.1 ± 74.0	225.5 ± 54.4	0.013
Albumin, g/L	40.9 ± 4.0	41.8 ± 4.2	42.2 ± 3.5	41.8 ± 3.4	0.171
Homocysteine	18.1 ± 9.2	16.2 ± 6.7	17.5 ± 7.1	18.2 ± 7.1	0.129
FPG, mg/dL	103.5 ± 19.2	117.4 ± 31.1	137.0 ± 37.6	200.0 ± 97.3	< 0.001
TC, mg/dL	147.1 ± 40.9	155.5 ± 44.2	167.4 ± 47.2	175.7 ± 49.2	< 0.001
TG, mg/dL	62.3 ± 18.2	101.6 ± 22.4	147.4 ± 41.6	293.0 ± 265.9	< 0.001
HDL-C, mg/dL	57.4 ± 17.3	49.6 ± 11.8	46.1 ± 10.9	37.8 ± 9.3	< 0.001
LDL-C, mg/dL	72.4 ± 29.1	85.7 ± 37.2	95.9 ± 39.5	98.6 ± 36.3	< 0.001
Uric acid, mg/dL	6.1 ± 1.9	6.1 ± 1.8	6.7 ± 1.8	6.9 ± 2.0	0.011
SCr, mg/dL	79.4 ± 42.1	85.8 ± 60.3	81.0 ± 32.7	81.9 ± 27.2	0.804
Coronary angiography					
Lesion vessels	0.7 ± 1.0	1.2 ± 1.2	1.4 ± 1.1	2.1 ± 1.0	< 0.001
Three-vessel disease	20 (24.7)	30 (37.5)	39 (49.4)	61 (76.3)	< 0.001
Gensini score, (IQR)	30.5 (20.5,43.5)	32.0 (20.0,44.0)	42.5 (29.0,61.0)	66.5 (51.0,97.1)	< 0.001
Medications use at discharge					
Antiplatelet	14 (17.3)	16 (20.0)	16 (20.3)	20 (25.0)	0.679
Statin	8 (9.9)	6 (7.5)	9 (11.4)	8 (10.0)	0.870
CCB	22 (27.2)	11 (13.8)	10 (12.7)	16 (20.0)	0.067
Beta blockers	11 (13.6)	4 (5.0)	9 (11.4)	7 (8.8)	0.289
ACEI/ARB	15 (18.5)	5 (6.3)	3 (3.8)	0 (0)	< 0.001

Data are presented as the IQR, mean ± SD or n (%)

*BMI* body mass index, *SBP* systolic blood pressure, *DBP* diastolic blood pressure, *IQR* interquartile range, *WBC* white blood cell, *FPG* fasting plasma glucose, *TC* total cholesterol, *TG* Triglycerides, *HDL-C* high-density lipoprotein cholesterol, *LDL-C* low-density lipoprotein cholesterol, *SCr* Serum creatinine concentration, *CCB* calcium channel blocker, *ACEI*, angiotensin-converting enzyme inhibitor, *ARB* angiotensin receptor blocker

### Relationship between TyG index and metabolic syndrome

**Fig. 1:**
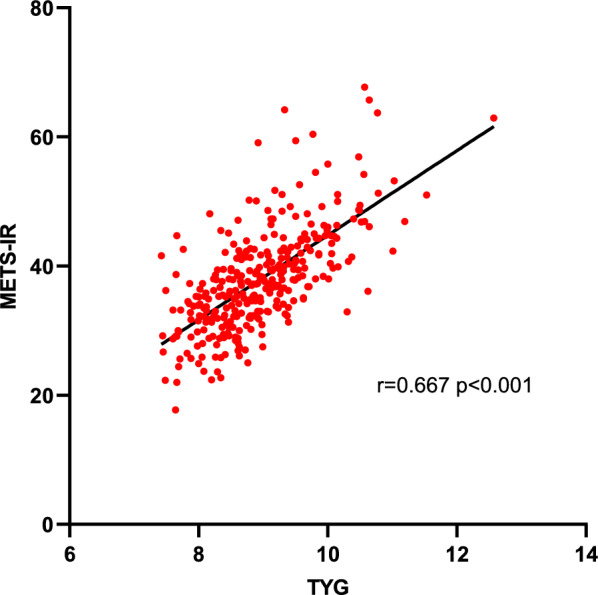
Correlation between TyG index and METS-IR index

**Fig. 2:**
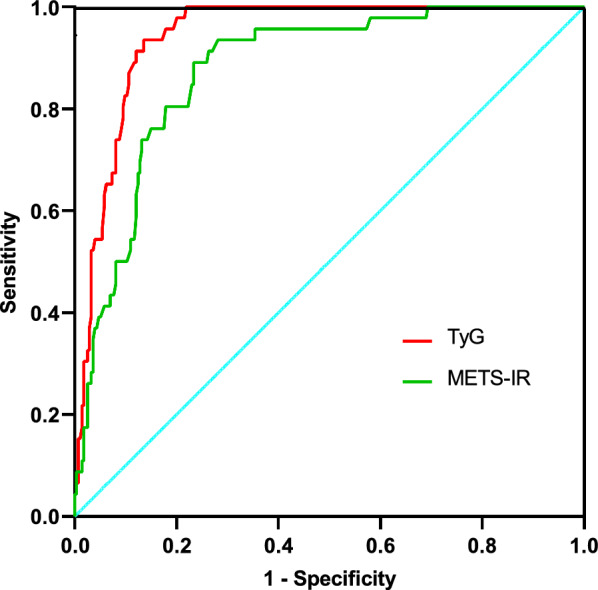
ROC curve of the TyG index and METS-IR in the detection of metabolic syndrome

A total of 46 patients, accounting for 14.4% of the sample, exhibited a combined metabolic syndrome. Notably, the TyG quartile group displayed a higher proportion of Q3 and Q4 metabolic syndrome cases, as indicated in Table 1. Furthermore, within the Q4 group (TyG range 9.42–12.57), there was a greater concentration of metabolic syndrome cases. The TyG index demonstrated a significant association with METS-IR, with a correlation coefficient of 0.667 (p < 0.001), as depicted in Fig. 1. Additionally, when predicting metabolic syndrome using the ROC curve, the area under the curve (AUC) for the TyG index (AUC = 0.943, 95% CI 0.918–0.967, p < 0.001) surpassed that of METS-IR (AUC = 0.876, 95% CI 0.829–0.924, p < 0.001), as illustrated in Fig. 2.

### Relationship between TyG index and the degree of coronary artery lesion stenosis

According to the baseline characteristics of the trichotomous subgroups of the Gensini score shown in Table 2, patients in the high stenosis group (Gensini score ≥ 53) had higher levels of TyG index, homocysteine, fasting glucose, leukocytes, neutrophil ratio and blood creatinine, a higher prevalence of diabetes mellitus and metabolic syndrome, more frequent use of cigarettes and alcohol, and fewer people treated with ACEI/ARB drugs (all p values < 0.05). In particular, the number of diseased vessels and triple lesions was significantly higher in the high stenosis group than in the low stenosis group (p < 0.001).

**Table 2:** Clinical and biological characteristics according to the Gensini score tertiles and degree of CAD

Variable	Gensini score tertiles				Degree of CAD				
	Lowest tertile ≤ 32 (n = 114)	Mid tertile 32 ~ 53 (n = 100)	Highest tertile ≥ 53 (n = 106)	p value	Non-severe stenosis (n = 214)	Sever stenosis (n = 106)		p value	
TyG index	8.47 ± 0.51	8.94 ± 0.69	9.48 ± 0.86	< 0.001	8.7 ± 0.6	9.5 ± 0.9		< 0.001	
Age, years	65.0 ± 10.6	67.0 ± 10.0	65.8 ± 10.3	0.165	67.3 ± 10.3	65.7 ± 10.6		0.181	
Male	53 (46.5)	54 (54.0)	55 (51.9)	0.521	107 (50.0)	55 (51.9)		0.751	
BMI, kg/m^2^	23.1 ± 3.1	24.4 ± 3.6	24.3 ± 2.7	0.005	23.7 ± 3.4	24.3 ± 2.7		0.136	
SBP, mmHg	141.2 ± 28.0	138.2 ± 20.1	139.8 ± 20.4	0.784	139.8 ± 24.6	139.8 ± 20.4		0.998	
DBP, mmHg	81.6 ± 15.4	82.5 ± 11.8	81.4 ± 11.4	0.567	82.0 ± 13.8	81.4 ± 11.4		0.698	
Heart rate, bpm	75.5 ± 15.0	77.0 ± 13.2	77.3 ± 13.9	0.536	76.2 ± 14.2	77.3 ± 13.9		0.521	
Smoker	9 (7.9)	30 (30.0)	49 (46.2)	< 0.001	39 (18.2)	49 (46.2)		< 0.001	
Drinker	2 (1.8)	7 (7.0)	19 (17.9)	< 0.001	9 (4.2)	19 (17.9)		< 0.001	
H-type hypertension	34 (29.8)	53 (53.0)	67 (63.2)	< 0.001	87 (40.7)	67 (63.2)		< 0.001	
METS-IR index	34.2 ± 6.0	38.5 ± 7.9	41.3 ± 8.1	0.067	36.2 ± 7.3	41.3 ± 8.1		< 0.001	
Metabolic syndrome	2 (1.8)	6 (6.0)	38 (35.8)	< 0.001	8 (3.7)	38 (35.8)		< 0.001	
Diabetes mellitus	20 (17.5)	33 (33.0)	42 (39.6)	0.001	53 (24.8)	42 (39.6)		0.006	
Biochemical indicators									
WBC, 10^9^/L	6.0 ± 2.0	6.5 ± 2.0	6.6 ± 2.2	0.065	6.3 ± 2.0		6.6 ± 2.2		0.130
Neutrophil ratio, %	65.8 ± 10.8	66.5 ± 9.6	66.3 ± 10.2	0.819	66.1 ± 10.2		66.6 ± 10.1		0.702
Platelet, 10^9^/L	211.2 ± 62.2	225.7 ± 65.9	211.3 ± 55.4	0.153	218.0 ± 64.2		211.3 ± 55.4		0.357
Albumin, g/L	41.6 ± 3.7	41.9 ± 3.6	41.6 ± 4.1	0.083	41.8 ± 3.7		41.6 ± 4.1		0.646
Homocysteine	16.4 ± 8.4	18.4 ± 8.1	17.8 ± 6.0	< 0.001	17.3 ± 8.3		17.8 ± 6.0		0.078
FPG,mg/dl	111.2 ± 29.3	140.4 ± 64.5	168.7 ± 82.2	< 0.001	124.8 ± 51.1		168.7 ± 82.2		< 0.001
TC, mg/dl	155.7 ± 42.8	158.5 ± 44.7	170.2 ± 51.1	0.051	157.0 ± 43.6		170.2 ± 51.1		0.017
TG, mg/dl	98.3 ± 47.8	1138.3 ± 87.2	219.0 ± 246.8	< 0.001	117.0 ± 71.7		219.0 ± 246.8		< 0.001
HDL-C, mg/dl	52.7 ± 15.0	47.5 ± 12.8	42.6 ± 13.7	< 0.001	50.3 ± 14.2		42.6 ± 13.7		< 0.001
LDL-C, mg/dl	83.3 ± 33.5	86.0 ± 36.4	95.2 ± 40.3	0.047	84.6 ± 34.8		95.2 ± 40.3		0.016
Uric acid, mg/dl	6.2 ± 1.8	6.3 ± 2.1	6.8 ± 1.8	0.047	6.2 ± 2.0		6.8 ± 1.8		0.015
SCr, umol/L	85.4 ± 49.9	77.7 ± 37.6	82.4 ± 37.7	0.064	81.8 ± 44.7		82.4 ± 37.7		0.908
Coronary angiography									
Lesion vessels	0.6 ± 1.0	1.1 ± 1.1	1.7 ± 1.2	< 0.001					
Three-vessel disease	28(24.6)	47(47.0)	75(70.8)	< 0.001					
Gensini score, (IQR)	23.5 ± 4.3	41.9 ± 5.8	88.5 ± 34.1	< 0.001					
Medication, n (%)									
Antiplatelet	23 (20.2)	18 (18.0)	25 (23.6)	0.606	41 (19.2)		25 (23.6)		0.357
Statin	10 (8.8)	10(10.0)	11 (104)	0.915	20 (9.3)		11 (10.4)		0.769
CCB	25 (21.9)	20 (20.0)	14 (13.2)	0.221	45 (21.0)		14 (13.2)		0.090
Beta blockers	11 (9.6)	7 (7.0)	13 (12.3)	0.443	18 (8.4)		13 (12.3)		0.273
ACEI/ARB	17 (14.9)	5 (5.0)	1 (0.9)	< 0.001	22 (10.3)		1 (0.9)		0.002

Data are presented as the IQR, mean ± SD or n (%)

*BMI* body mass index, *SBP* systolic blood pressure, *DBP* diastolic blood pressure, *IQR* interquartile range, *hs-CRP* hypersensitive C-reactive protein, *WBC* white blood cell, *FPG* fasting plasma glucose, *TC* total cholesterol, *TG* Triglycerides, *HDL-C* high-density lipoprotein cholesterol, *LDL-C* low-density lipoprotein cholesterol, *SCr* Serum creatinine concentration, *CCB* calcium channel blocker, *ACEI*, angiotensin-converting enzyme inhibitor, *ARB* angiotensin receptor blocker

### Relationship between H-type hypertension and the degree of coronary artery lesion stenosis

As shown in Table 2, the degree of coronary stenosis and the likelihood of severe stenosis were significantly increased in patients with H-type hypertension compared to non-H-type hypertension (p < 0.001). The correlation between TyG index and Gensini score was significantly higher in patients with H-type hypertension than in patients without H-type hypertension.

### Correlation of risk factors with gensini score in H-type and non-H-type hypertension groups

The Gensini scores and risk factors were independently correlated in each of the groups, as presented in Table 3. In the cohort of individuals with H-type hypertension, several factors including BMI, FBP, TG, LDL-c, and METS-IR exhibited a positive correlation with the Gensini score, surpassing the levels observed in the non-H-type hypertension group (all p < 0.01). Conversely, HDL-c demonstrated a negative association (r = − 0.408, p < 0.001). Furthermore, the TyG index displayed a significantly stronger correlation with the Gensini score in the H-type hypertensive group (r = 0.766, p < 0.001) compared to the non-H-type hypertensive group (r = 0.250, p < 0.001).

**Table 3:** Correlation between gensini scores and risk factors of two groups

Variable	H-type hypertension		Non- H-type hypertension	
	Correlation coefficient (r)	P value	Correlation coefficient (r)	P value
TyG index	0.766	< 0.001	0.250	0.001
BMI, kg/m^2^	0.240	0.003	0.082	0.291
FPG,mg/dl	0.442	< 0.001	0.245	0.001
TG, mg/dl	0.297	< 0.001	0.180	0.021
HDL-C, mg/dl	− 0.408	< 0.001	− 0.167	0.032
LDL-C, mg/dl	0.254	0.001	− 0.006	0.936
Uric acid, mg/dl	0.211	0.008	0.011	0.893
METS-IR index	0.428	< 0.001	0.159	0.041

### TyG index and coronary lesion severity and predictive value

The prevalence of diabetes, metabolic syndrome, smoking history, and alcohol consumption history exhibited a significant increase in the severe stenosis group compared to the non-serious stenosis group (p < 0.001) (Table 2). Conversely, the usage of ACEI/ARB class drugs displayed a significant decrease in the severe stenosis group compared to the non-serious stenosis group (p < 0.001). Additionally, statistically significant differences were observed between the severe stenosis and non-severe stenosis groups in terms of TyG index, FBG, WBC, albumin, and blood creatinine (p < 0.05). The number of coronary lesions (p < 0.001) and the degree of stenosis (p = 0.02) exhibited a positive correlation with TyG index values (Table 1). Furthermore, the incidence of three lesions and severe stenosis was higher in the TyG index quartile Q4 subgroup compared to the Q1 subgroup (50.0 vs. 25.6, p = 0.021).

The present study employed multinomial logistic regression analyses (Table 4) to examine the associations between TyG, degree of stenosis, and number of diseased vessels. The TyG index demonstrates a significant association with severe coronary stenosis, as evidenced by an odds ratio (OR) of 7.094 (95% confidence interval CI 4.801–10.484, p < 0.0001). Furthermore, a significant association between the TyG index and multivessel disease is observed, with an OR of 3.982 (95% CI 2.648–5.990, p < 0.0001). Following the appropriate adjustment for notable factors linked to coronary stenosis, including diabetes, smoking, and other relevant variables, it was observed that the TyG index remained significantly associated with an elevated likelihood of having diseased vessels (odds ratio [OR] 1.862, 95% confidence interval CI 1.036–3.348, p-value 0.05). Additionally, the TyG index was found to be strongly correlated with an increased prevalence of coronary stenosis (OR 4.000, 95% CI 2.411–6.635, p-value 0.0001).

**Table 4:** Correlation between TyG index and number of diseased vessels and degree of stenosis

Unadjusted OR (95% CI)		p value	Model 1 OR (95% CI)	p value	Model 2 OR (95% CI)	p value
Number of vessels with stenosis						
0	1		1		1	
1	2.032 (1.247–3.311)	0.004	1.160 (0.634–2.123)	0.630	1.476 (0.725–3.005)	0.283
2 or 3	3.982 (2.648–5.990)	< 0.0001	1.855 (1.118–3.077)	0.017	1.862 (1.036–3.348)	0.038
Degree of coronary stenosis						
Low	1		1			
Mid	3.000 (2.048–4.393)	< 0.0001	2.610 (1.675–4.067)	< 0.0001	2.053 (1.248–3.379)	0.005
Sever	7.094 (4.801–10.484)	< 0.0001	4.379 (2.798–6.854)	< 0.0001	4.000 (2.411–6.635)	< 0.0001

Multinomial logistic regression analyses were performed

*Model 1* Adjusted for diabetes mellitus, metabolic syndrome, smoking, *Model 2* Model 1 + adjusted for METS-IR

**Fig. 3:**
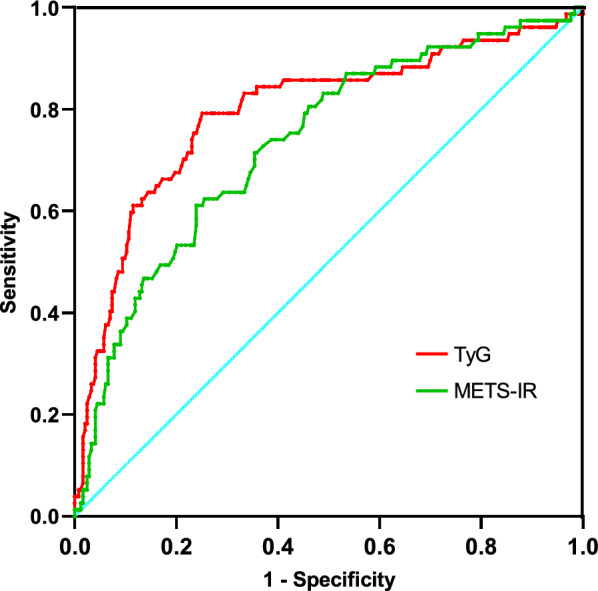
Receiver operating characteristic (ROC) curves for the TyG index and METS-IR index, both of which serve as predictive measures of severe stenosis in patients diagnosed with H-type hypertension

**Fig. 4:**
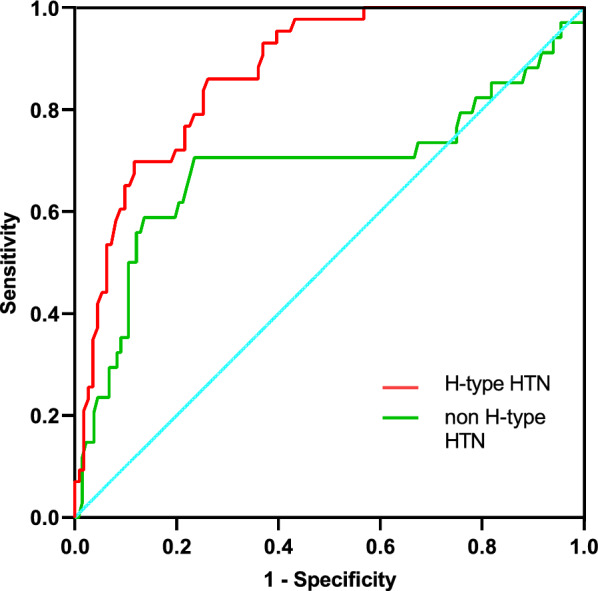
AUCs of the TyG index in predicting the development of severe coronary artery lesions in patients with and without H-type hypertension

**Fig. 5:**
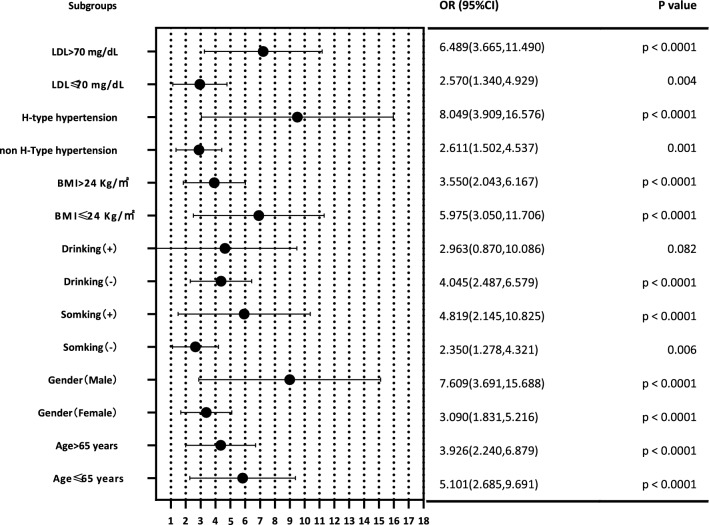
Subgroup analyses of the effect of the TyG index on the incidence of severe stenosis. Each subgroup analysis was adjusted for age, sex, BMI, smoking, alcohol consumption, LDL-C, H-type hypertension, if not stratified. TyG triglyceride glucose, *BMI* body mass index, *LDL-C* low-density lipoprotein cholesterol, *CI* confidence interval

Figure 3 illustrates the Receiver Operating Characteristic (ROC) curves pertaining to the TyG index and METS-IR index, both of which serve as predictive measures for severe stenosis in patients diagnosed with H-type hypertension. At a TyG index threshold of 9.13, the receiver operating characteristic (ROC) curve yielded an area under the curve (AUC) of 0.780 (95% confidence interval CI 0.722–0.838, p < 0.0001). The sensitivity and specificity of the test were determined to be 73% and 52%, respectively. Moreover, the TyG index exhibited a relatively higher level of effectiveness in comparison to other indices, namely the METS-IR index and Hcy and TG, as demonstrated in Table 5. And the TyG index predicts the development of severe coronary stenosis in patients with H-type hypertension better than in those without (Fig. 4). The results of subgroup analyses revealed notable disparities in the prevalence of severe coronary stenosis across different demographic and clinical groups. TyG index was associated with an increased prevalence of developing severe coronary lesions in the subgroups of smokers and LDL > 70 mg/dL, age ≤ 65 years, and BMI ≤ 24. Specifically, it was observed that males exhibited a significantly higher prevalence of severe coronary stenosis compared to females. Furthermore, patients diagnosed with H-type hypertension exhibited a significantly higher prevalence of severe coronary stenosis when compared to their counterparts without this condition (refer to Fig. 5 for detailed findings).

**Table 5:** AUCs of TG, Hcy, TyG index and METS-IR index predicting the occurrence of sever CAD

Variable	AUC(95%CI)	P value
TG	0.732 (0.665,0.798)	< 0.001
METS-IR index	0.733 (0.668,0.798)	< 0.001
TyG index	0.795 (0.731,0.858)	< 0.001
Hcy	0.509 (0.440,0.577)	0.821

## Disscusion

In the current investigation, a cohort of patients diagnosed with H-type hypertension was incorporated to explore the potential of the TyG index as a predictive tool for the emergence of severe stenosis in H-type hypertension. Our study has made a novel discovery by demonstrating a positive correlation between elevated levels of TyG and an increased likelihood of severe stenosis in individuals with H-type hypertension. Furthermore, we have accounted for potential confounding factors, such as smoking and the METS-IR index, and have found a significant association between the TyG index and the risk of severe stenosis in H-type hypertension patients (odds ratio [OR] 4.000, 95% confidence interval CI 2.411–6.635, p < 0.0001). Additionally, we have observed a similar association between the TyG index and the presence of multivessel disease (OR 1.862, 95% CI 1.036, 3.348, p 0.001). Notably, our analysis of the receiver operating characteristic (ROC) curve indicates that the TyG index exhibits a favorable predictive value for the development of severe stenosis in patients with H-type hypertension. Furthermore, it possesses the potential to serve as a prognosticator for metabolic syndrome.

Insulin resistance (IR) is characterized by the compromised functionality and impaired regulation of insulin-mediated glucose metabolism within various tissues, representing an aberrant physiological condition [31]. This state serves as one of the initial indications of the onset of type 2 diabetes mellitus (T2DM) and cardiovascular ailments [32]. Dysregulation of glucose and lipid metabolism is considered an important factor in the pathogenesis and aetiology of type 2 diabetes mellitus (T2DM) [33, 34], which also plays a key role in the development and progression of CAD [35]. Numerous studies have demonstrated that the TyG index is a valuable indicator of a simple and effective predictor of coronary heart disease risk [36]. An elevated TyG index is associated with greater odds of coronary stenosis [37], plaque progression [24], and more vascular lesions [38], which is consistent with our findings. Thai et al. found that an increase in the TyG index identifies patients at high risk of coronary artery stenosis and correlates with the number and severity of stenoses [39]. A dose–response relationship has also been observed between the TyG index and the severity of coronary heart disease [38]. We also found that the TyG index, as a combined index based on glucose and TG levels, was more effective and sensitive in predicting the development of severe coronary artery disease in H-type hypertensive patients compared with the use of glucose and TG alone, which is in line with the findings of Zhao et al. [39–43]. The TyG index holds significant relevance in the evaluation of both type 2 diabetes mellitus (T2DM) and cardiovascular disease (CVD), and it additionally assumes a pivotal role in the context of CVD [17, 44].

The TyG index has been identified as a reliable and valuable tool for evaluating the presence of type 2 diabetes and metabolic syndrome, as supported by previous research studies [45, 46]. An elevated TyG index is positively correlated with the severity of glucose and lipid metabolism disorders, as well as the increased prevalence of metabolic syndrome in patients. Consequently, the TyG index holds significant value as a predictive tool for metabolic syndrome, as evidenced by its high area under the curve (AUC) of 0.924 (95% CI 0.905–0.943, p < 0.001). In a similar vein, the TyG index has been utilized to prognosticate non-alcoholic fatty liver disease (NAFLD) [47], ischemic stroke [48], atrial fibrillation [49], carotid atherosclerosis [50], as well as the onset of diseases within the realm of oncology [51] and chronic kidney disease (CKD) [52]. The present study aimed to investigate the association between the TyG index and the conventional etiology of cardiovascular disease. Intriguingly, our findings revealed a significant negative correlation between the TyG index and age, aligning with prior research conducted by Zhao et al. [40, 53]. In our study, we discovered a significant association between older age and lower levels of triglycerides, which played a significant role in contributing to this effect.

Hypertension and diabetes play a synergistic role in cardiovascular disease, and control of blood pressure and blood glucose, as important risk factors for cardiovascular disease, is critical in the secondary prevention of cardiovascular. The interplay between hypertension and diabetes is known to exert a synergistic effect on the development and progression of cardiovascular disease. Given their significance as key risk factors, effective management of both blood pressure and blood glucose assumes paramount importance in the context of secondary prevention strategies for cardiovascular disease [54]. The TyG index has been consistently demonstrated in numerous studies as an effective predictor of both prognosis and risk associated with cardiovascular disease [25, 53]. The majority of investigations conducted thus far have primarily focused on diabetic individuals, leaving a dearth of research pertaining to the prognostication of coronary artery disease severity in hypertensive patients, as well as the evaluation of the correlation between atherosclerosis, hyperuricemia, and stroke in patients afflicted with H-type hypertension [41, 55, 56]. The present study provides novel evidence indicating that the TyG index exhibits promising predictive capabilities in assessing the severity of coronary artery disease (CAD) among individuals diagnosed with H-type hypertension. This finding represents a significant contribution to the existing body of knowledge in this field. The incidence of severe coronary stenosis exhibited a statistically significant elevation in patients diagnosed with H-type hypertension, as compared to patients without H-type hypertension. Nevertheless, the precise mechanism underlying the association between H-type hypertension and coronary artery disease (CAD) remains largely elusive, necessitating the need for additional investigations to validate this relationship.

In our subgroup analysis, it was observed that the incidence of severe stenosis was found to be significantly higher in the male population as compared to the female population. This disparity may be attributed to the higher prevalence of risk factors associated with the development and progression of cardiovascular disease among men, including but not limited to unhealthy lifestyle behaviors such as smoking and alcohol consumption. Specifically, the prevalence of these risk factors was found to be 32.7% in men compared to 22.2% in women for smoking, and 11.7% in men compared to 5.7% in women for alcohol consumption. Males exhibit a propensity for heightened stress levels in comparison to females, and it is plausible that the presence of life stressors may serve as a contributing factor to this observed disparity [57]. We also found a stronger interaction of smoking and elevated LDL with TyG index, and a stronger interaction of BMI ≤ 24 with TyG index compared to BMI > 24. This may be related to the obesity paradox, where mild obesity, especially overweight, is associated with improved survival [58], which needs to be confirmed by further relevant studies.

The present investigation exhibits certain inherent limitations. This study is of a retrospective nature, thereby precluding the establishment of a definitive causal relationship between the TyG index and severe stenosis. Second, the underlying mechanism governing the progression of the TyG index and coronary artery disease (CAD) remains inadequately elucidated. Third, it is imperative to note that the subjects included in this study were derived exclusively from a singular region and exhibited a limited sample size. Consequently, it is crucial to validate these findings through a comprehensive multi-center and multi-regional study encompassing a substantial sample size. Additionally, it is important to acknowledge that solely the initial laboratory test results obtained upon admission were collected, and only subjects with a diagnosis of CAD were included in this study, potentially introducing certain selection biases. Hence, it is imperative to conduct further multicenter and prospective investigations to corroborate these observations.

## Conclusion

In summary, our findings suggest that the TyG index serves as a reliable indicator for evaluating the extent of coronary artery disease (CAD) in individuals with H-type hypertension. Moreover, it emerges as an independent prognostic factor for both the severity of CAD and the presence of metabolic syndrome. Notably, a noteworthy association is observed between the TyG index and the number of coronary stenosis as well as the involvement of coronary vessels in the development of lesions. The TyG index presents itself as a viable and cost-effective biological index that holds potential for implementation across a diverse array of primary healthcare facilities within China. Its utilization can prove instrumental in the process of risk stratification and intervention, thereby mitigating the occurrence of unfavorable cardiovascular events among patients afflicted with H-type hypertension.

## Data Availability

The datasets used and/or analyzed in the study are available from the cor- responding author upon reasonable request.

## Abbreviations

IR: Insulin resistance
TyG index: Triglyceride–glucose index
Mets: Metabolic syndrome
CVD: Cardiovascular disease
HTN: Hypertension
BMI: Body mass index
SBP: Systolic blood pressure
DBP: Diastolic blood pressure
IQR: Interquartile range
WBC: White blood cell
FPG: Fasting plasma glucose
TC: Total cholesterol
TG: Triglycerides
HDL-C: High-density lipoprotein cholesterol
LDL-C: Low-density lipoprotein cholesterol
SCr: Serum creatinine concentration
Hcy: Homocysteine
CCB: Calcium channel blocker
ACEI: Angiotensin-converting enzyme inhibitor
ARB: Angiotensin receptor blocker
